# Maternal common mental disorder symptoms and child nutritional outcomes: The role of caregiving practices among children aged 6–59 months

**DOI:** 10.4102/phcfm.v18i1.5369

**Published:** 2026-09-15

**Authors:** Anesu Marume, Moherndran Archary, James January, Saajida Mahomed

**Affiliations:** 1 School of Nursing and Public Health, College of Health Sciences, University of KwaZulu-Natal, Durban, South Africa; 2 Department of Global Public Health and Family Medicine, Faculty of Medicine and Health Sciences, University of Zimbabwe, Harare, Zimbabwe; 3 Department of Paediatrics and Child Health, Victoria Mxenge Hospital, University of KwaZulu-Natal, Durban, South Africa; 4 Department of Psychiatry and Mental Health, Faculty of Medicine and Health Sciences, Kamuzu University of Health Sciences, Blantyre, Malawi; 5 School of Laboratory Medicine and Medical Sciences, College of Health Sciences, University of KwaZulu-Natal, Durban, South Africa

**Keywords:** common mental disorders, depression, anxiety, malnutrition, stunting, primary health care

## Abstract

**Background:**

Maternal common mental disorders (CMDs) remain under recognised in many low-income countries, and these can compromise caregiving capacity during early childhood. Evidence linking maternal CMD symptoms to caregiving practices and child nutritional outcomes in Zimbabwe and similar settings remains limited.

**Aim:**

This study examined associations between maternal CMD symptoms, caregiving practices and nutritional outcomes among young children in Zimbabwe.

**Setting:**

The study was conducted in the Manicaland and Matabeleland South provinces of Zimbabwe.

**Methods:**

A secondary analysis of data from a household-based matched case-control study involving mother–child dyads attending primary health facilities was conducted. Maternal CMD symptoms were assessed using the Shona Symptom Questionnaire (SSQ 14) and analysed as a continuous variable. Multivariable logistic regression models were used to examine associations between maternal CMD symptoms and caregiving and child nutritional outcomes, adjusting for maternal, child and household covariates.

**Results:**

The prevalence of probable maternal CMD was 34.0% (95% confidence interval: 29.4–38.9). Higher maternal CMD symptom scores were associated with lower odds of antenatal care attendance, incomplete child vaccination and reduced uptake of vitamin A supplementation. Higher CMD symptoms were also associated with increased odds of early weaning, childhood stunting and childhood wasting.

**Conclusion:**

Maternal CMD symptoms were associated with diminished caregiving practices and adverse child nutritional outcomes. Integrating maternal mental health screening and support into routine maternal and child health services may improve child health and nutrition outcomes.

**Contribution:**

This study provides evidence linking maternal CMD symptoms with caregiving practices and child nutritional outcomes in a low-resource setting where such evidence remains limited.

## Introduction

Common mental disorders (CMDs) refer to non-psychotic mental health conditions, such as depression, anxiety and somatoform disorders.^1^ These disorders are among the leading causes of disability worldwide.^2^ Women are generally about twice as likely as men to experience CMDs.^3^ The prevalence is particularly high during the perinatal period, starting from pregnancy through the first year postpartum, when expectations and responsibilities increase and predispose women to depression and anxiety symptoms.^4^ The burden of maternal CMDs tends to peak during late pregnancy and early postpartum, with symptoms often declining as the child grows older.^5,6,7^

Maternal CMDs are consistently more prevalent in low- and middle-income countries (LMICs) than in high-income countries. This disparity reflects the greater exposure of women in LMICs to social and economic adversities, including poverty, food insecurity, intimate partner violence, limited social support and high caregiving burdens.^8,9,10^ Structural factors such as gender inequality, unplanned pregnancies and reduced access to quality perinatal and mental health services further compound vulnerability.^11,12^ The burden of CMDs is higher among women with prior psychiatric history or chronic stressors during the perinatal period.^13,14^

During early childhood, an infant depends almost entirely on a primary caregiver, usually the mother, for survival, nutrition and stimulation.^15^ Maternal CMDs may impair maternal responsiveness and caregiving capacity, increasing the likelihood of delayed breastfeeding initiation, suboptimal milk production, early introduction of solid foods and inadequate attention to the child, all of which increase the risk of poor growth and development. Evidence also suggests a bidirectional relationship between maternal CMDs and child malnutrition.^16^ Maternal psychological distress may contribute to poor child nutrition, and at the same time, having a malnourished or frequently ill child may, in turn, exacerbate maternal CMD symptoms.^17^

Globally, undernutrition is a contributing factor in over half of the deaths among children under 5 years.^18^ Stunting, a key indicator of chronic undernutrition, is particularly prevalent in LMICs, where 43% of all stunted children reside in Africa.^16^ Although extensive research has investigated the effects of diet, comorbidities, household characteristics and water and sanitation on child nutrition,^19,20^ there remains a paucity of evidence on the contribution of maternal CMDs to child stunting, especially in LMICs.

Despite growing recognition of the burden of maternal CMDs in low- and middle-income countries, important evidence gaps remain. Most studies from sub-Saharan Africa have examined maternal mental health in relation to single-child outcomes, most commonly stunting, without simultaneously considering broader caregiving practices and preventive health behaviours that shape early childhood nutrition and development. Evidence from Zimbabwe and similar settings remains limited, particularly studies using locally validated mental health screening tools such as the Shona Symptom Questionnaire. Fewer analyses have explored maternal CMD symptoms as a continuous construct, which better reflects the dimensional nature of psychological distress and avoids information loss associated with dichotomisation. Lastly, the potential for bidirectional relationships between maternal psychological distress and child nutritional outcomes is often acknowledged but seldom examined in applied analyses.

Addressing these gaps is critical for informing integrated maternal mental health and child nutrition interventions within primary health care systems. This study, therefore, sought to examine associations between maternal CMD symptoms, caregiving practices and nutritional outcomes among young children in Zimbabwe.

## Research methods and design

### Study design and data source

This study was a secondary analysis of data from a household-based matched case-control study conducted in the Manicaland and Matabeleland South provinces of Zimbabwe. The parent study enrolled caregiver–child pairs using a 1:2 matched design to investigate determinants of child nutritional outcomes. Detailed procedures of the parent study have been described elsewhere.^21^ For the purposes of the present analysis, we focused specifically on the association between maternal CMD symptoms, caregiving practices and child nutritional outcomes.

### Study setting

The study was conducted in Manicaland and Matabeleland South provinces, predominantly rural regions of Zimbabwe characterised by high levels of food insecurity, limited access to specialised mental health services and a high burden of childhood undernutrition.

### Study population and analytic sample

The parent dataset comprised 450 caregiver–child pairs. For this secondary analysis, we restricted the sample to biological mother–child dyads in which the mother completed the Shona Symptom Questionnaire (SSQ-14). Caregivers who were not the child’s biological mother or had missing SSQ-14 data (*n* = 53) were excluded. The final analytic sample, therefore, consisted of 397 mother–child dyads.

Although the original study applied matching, the current analysis did not retain the matched structure. Matching was broken because of the exclusion of non-maternal caregivers and missing mental health data. Accordingly, standard regression modelling was applied, with adjustment for relevant maternal, child and household covariates to account for potential confounding.

### Measurement of maternal common mental disorder symptoms

Maternal CMD symptoms were assessed using the SSQ-14, a locally developed and validated screening tool widely used in Zimbabwe.^22^ The SSQ-14 consists of 14 binary (‘yes/no’) items capturing symptoms of depression, anxiety, somatic distress and psychotic experiences over the preceding 7 days.^21^

### Outcome variables

Outcomes were grouped into child nutritional outcomes and caregiving and health-service utilisation indicators. Anthropometric indicators were defined using the World Health Organization child growth standards. A child was considered stunted if their height-for-age Z-score was below −2 standard deviations (s.d.), wasted if their weight-for-height Z-score was below −2 s.d. and overweight if their weight-for-height was above +2 s.d.

Caregiving and health services indicators included:

- Early complementary feeding, defined as the introduction of solid or semi-solid foods prior to 6 months of age.
- Early weaning, characterised by the cessation of breastfeeding before 12 months of age.
- Low meal frequency, specified as fewer than three meals per day among children aged 6 months or older.
- Antenatal care (ANC) attendance, denoting at least one ANC visit during pregnancy.
- Health facility delivery.
- A child was termed fully vaccinated if they had an up-to-date record of vaccinations appropriate for their age.
- Child vitamin A supplementation uptake within the previous 6 months.
- Maternal receipt of iron-folate supplementation during pregnancy.
- Any episode of diarrhoea in the child within the 2 weeks prior to the survey.

### Covariates

Potential confounders were selected *a priori* based on existing literature and included maternal age, maternal education, maternal occupation, partner education, partner occupation, household asset index, marital status, parity, number of siblings, sibling age spacing, child age, child sex, child human immunodeficiency virus (HIV) status and birthweight history. Unless otherwise stated, all covariates were included in each multivariable model to ensure consistency across analyses.

### Data analysis

Descriptive statistics were used to summarise participant characteristics. Means and s.d. were reported for continuous variables, while frequencies and percentages were used for categorical variables.

Associations between maternal CMD symptoms and caregiving and nutritional outcomes were examined using multivariable logistic regression models, with the SSQ-14 total score entered as a continuous exposure. To calculate the prevalence of probable CMD, a score of ≥ 7 was adopted from the validation study in Zimbabwe.^20^ For all regression analyses, maternal CMD symptoms were treated as a continuous variable, represented by the total SSQ-14 score (range: 0–14). This approach preserves information on symptom severity and avoids misclassification associated with dichotomisation.^23^ This approach is consistent with methodological literature demonstrating that depressive and anxiety symptoms exist along a continuum rather than as discrete categories, and that continuous modelling improves statistical power while reducing misclassification bias.^24,25^ Results are presented as adjusted odds ratios (AORs) with 95% confidence intervals (95% CI). Statistical significance was set at *p* < 0.05. All analyses were conducted using R Studio (version 4.4.2).

### Ethical considerations

Permissions were granted by the Zimbabwe Ministry of Health and Child Care. Research participants signed a consent form after explanations of the study procedure in their preferred language. No personal identification details were collected during data collection. The Biomedical Research Ethics Committee at the University of KwaZulu-Natal (BE109/19) and the Medical Research Council of Zimbabwe (MRCZ/A/2479) provided ethical clearance for the study.

## Results

### Participant characteristics

Table 1 presents the socio-demographic, household and child characteristics of the 397 biological mother–child dyads included in this secondary analysis. The mean maternal age was 26.86 years (s.d.: 7.14), while the mean child age was 17.82 months (s.d.: 12.80), and the mean partner age was 32.61 years (s.d.: 8.53). Most mothers (65.00%) and their partners (79.10%) had attained at least a high school level of education, and over half of the mothers (58.40%) were unemployed. Among children, 32.50% were stunted, 8.60% were wasted, 21.91% were overweight and 5.00% were living with HIV. Nearly half of households (48.36%) were within the middle household asset index category.

**TABLE 1:** Socio-demographic, household and child characteristics of mother–child dyads (*N* = 397).

Variable	Classification	Total	
		*n*	%
Maternal occupation	Formal	50	12.60
	Informal	115	29.00
	Unemployed	232	58.40
Maternal education	Less than high school	139	35.00
	High school and above	258	65.00
Spousal occupation	Formal	154	38.80
	Informal	113	28.50
	Unemployed	130	32.70
Spousal education	Less than high school	83	20.90
	High school and above	314	79.10
Marital status	Cohabiting	34	8.60
	Married	301	75.80
	Single	62	15.60
Child’s sex	Female	183	46.10
	Male	214	53.90
Child’s HIV status	Negative	377	95.00
	Positive	20	5.00
Child anthropometrics	Wasted	34	8.60
	Overweight	87	21.91
	Stunted	129	32.50
Child’s birth outcomes	Low birth weight	80	20.20
	Short stature	71	17.70
Household asset index	High	53	13.35
	Low	152	38.29
	Middle	192	48.36
Siblings’ age difference	24 months and above	54	13.60
	Less than 24 months	277	69.80
	Only child	66	16.60

HIV, human immunodeficiency virus.

### Maternal common mental disorder symptoms

The prevalence of probable maternal CMD was 34.0% (95% CI: 29.4–38.9), with a mean SSQ-14 score of 4.52 (s.d.: 4.49). Anxiety-related symptoms were the most frequently reported. Nearly half of mothers (49%) reported excessive worrying or overthinking, 47% reported difficulty sleeping, 42% experienced nightmares and 41% reported problems with concentration. Depressive symptoms were also common, with 43% reporting that they found life very difficult and 27% reporting persistent unhappiness. Somatic symptoms included persistent tiredness (33%) and frequent stomach pain (31%). Psychotic symptoms, assessed through reports of hearing or seeing things that others do not, were reported by 17% of participants (Figure 1).

**FIGURE 1:**
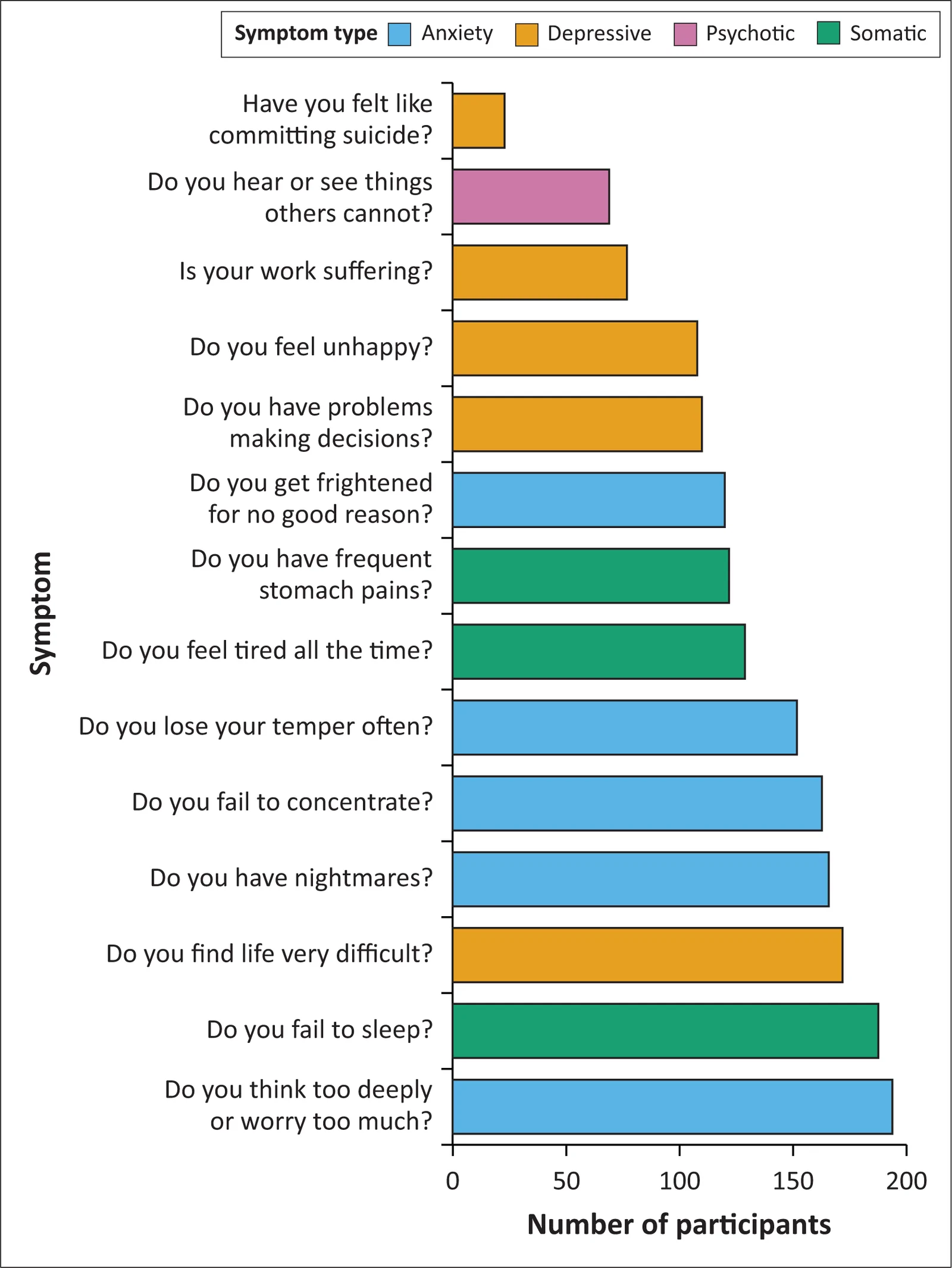
Frequency of maternal common mental disorder symptoms reported on the Shona Symptom Questionnaire.

### Factors associated with maternal common mental disorder symptom scores

Several maternal, child, and household factors were associated with maternal CMD symptom scores (Table 2). Increasing child age was positively associated with higher CMD scores (*β* = 0.015; 95% CI: 0.002–0.028). Higher maternal education (*β* = −0.550; 95% CI: −1.018 to −0.082) and higher spousal education (*β* = −1.267; 95% CI: −1.684 to −0.850) were associated with lower CMD symptom scores. Mothers engaged in informal employment (*β* = 1.299; 95% CI: 0.826–1.771) and those who were unemployed (*β* = 0.801; 95% CI: 0.184–1.418) reported higher CMD symptom scores, as did mothers whose partners were unemployed (*β* = 0.703; 95% CI: 0.249–1.157). Higher parity was associated with increased CMD symptom scores (*β* = 0.408; 95% CI: 0.101–0.714). Having a child who was HIV-infected, wasted or stunted, as well as a history of low birth weight, was also associated with higher maternal CMD symptom scores.

**TABLE 2:** Factors associated with maternal common mental disorder symptom scores.

Variable	Maternal CMD symptoms	
	*β*	95% CI
**Maternal age (years)**	−0.038	−0.108, 0.033
**Spousal age (years)**	0.006	−0.074, 0.085
**Child’s age (months)**	**0.015**	**0.002, 0.028**
**Sex**		
Male	−0.060	−1.049, 0.928
**Maternal education level**		
High school and above	**−0.550**	**−1.018, −0.082**
**Spousal education level**		
High school and above	**−1.267**	**−1.684, −0.850**
**Maternal occupation**		
Informal	**1.299**	**0.826, 1.771**
Unemployed	**0.801**	**0.184, 1.418**
**Spousal occupation**		
Informal	0.733	−0.156, 1.622
Unemployed	**0.703**	**0.249, 1.157**
**Marital status**		
Married	0.107	−1.244, 1.458
Cohabiting	−0.097	−1.861, 1.668
**Siblings age difference**	−0.012	−0.058, 0.035
**Household asset index**		
Low	0.825	−0.074, 1.724
Middle	0.534	−0.923, 1.991
Parity	**0.408**	**0.101, 0.714**
**Child’s HIV status**		
Infected	**0.993**	**0.129, 1.856**
**Wasting**		
Yes	**0.984**	**0.235, 1.732**
**Stunting**		
Yes	**1.064**	**0.193, 1.935**
**Overweight**		
Yes	0.301	−0.417, 1.018
**Birth weight**		
Low birth weight	**0.954**	**0.070, 1.837**
**Birth height**		
Short for gestation height	0.211	−1.427, 1.848

Note: Data in bold is statistically significant.

CMD, common mental disorder; CI, confidence interval; HIV, human immunodeficiency virus.

### Associations between maternal common mental disorder symptoms and caregiving, health-service utilisation and child nutritional outcomes

Figure 2 summarises the associations between maternal CMD symptom scores and caregiving practices, health-service utilisation and child nutritional outcomes. Higher maternal CMD symptom scores were associated with lower odds of ANC attendance (AOR = 0.55; 95% CI: 0.21–0.88), lower likelihood of children being up to date with vaccinations (AOR = 0.88; 95% CI: 0.71–0.97) and reduced uptake of vitamin A supplementation (AOR = 0.46; 95% CI: 0.11–0.70). No statistically significant associations were observed between maternal CMD symptoms and iron-folate supplementation during pregnancy or delivery at a health facility.

**FIGURE 2:**
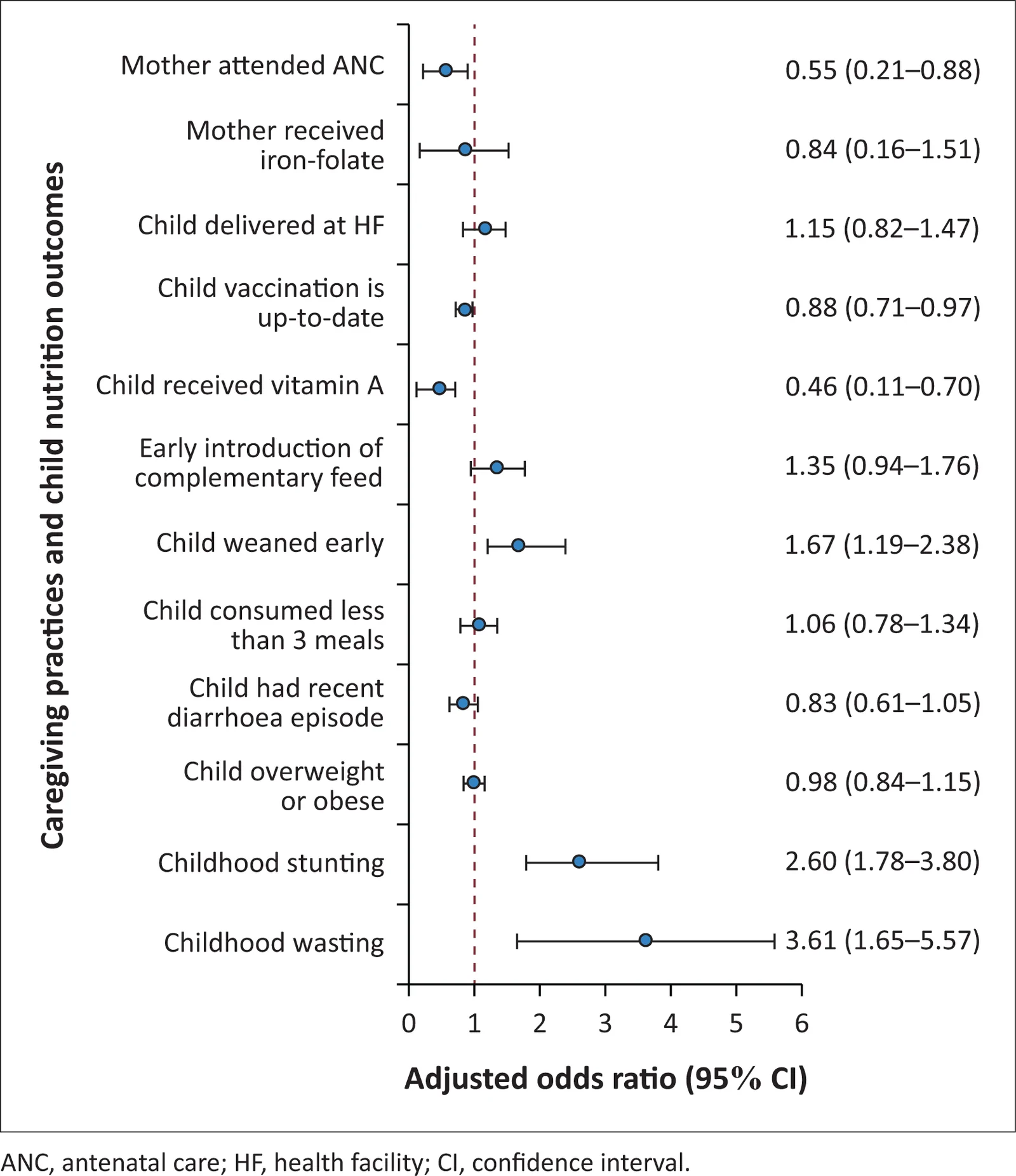
Associations between maternal common mental disorder symptom scores and caregiving practices and child nutritional outcomes.

Regarding child-feeding practices, higher CMD symptom scores were associated with increased odds of early weaning (AOR = 1.67; 95% CI: 1.19–2.38). Positive associations with early introduction of complementary feeding and low meal frequency did not reach statistical significance.

Higher maternal CMD symptom scores were strongly associated with childhood stunting (AOR = 2.60; 95% CI: 1.78–3.80) and childhood wasting (AOR = 3.61; 95% CI: 1.65–5.57). No significant association was observed between maternal CMD symptoms and child overweight or obesity. Maternal CMD symptom scores were not significantly associated with reported recent childhood diarrhoeal episodes (AOR = 0.83; 95% CI: 0.61–1.05).

## Discussion

Maternal CMD symptoms were prevalent in this population, affecting approximately one-third of mothers. Higher CMD symptom scores were associated with early complementary feeding, early weaning, reduced meal frequency, lower uptake of ANC, incomplete child vaccination and lower likelihood of vitamin A supplementation. In addition, higher maternal CMD symptoms were associated with increased odds of childhood stunting and wasting. These findings highlight the close co-occurrence of maternal psychological distress, caregiving capacity and child growth outcomes in a resource-constrained setting.

The observed prevalence of maternal CMD symptoms is consistent with findings from other LMICS, where perinatal and postnatal psychological distress remains common as a result of poverty, food insecurity, limited social support and high caregiving demands.^12^ Previous studies in South Asia and sub-Saharan Africa have documented associations between maternal depression or anxiety symptoms and suboptimal infant feeding practices, reduced utilisation of preventive health services and impaired child growth.^26,27^

The current study aligns with a growing body of evidence from LMICs showing that maternal CMDs are closely intertwined with household socioeconomic disadvantage, caregiving behaviours and child nutritional outcomes.^28^ Previous studies have documented similar patterns, whereby lower maternal and partner education levels and household economic insecurity are associated with higher maternal CMDs.^29,30^ Maternal CMDs frequently co-occur with suboptimal feeding practices, reduced uptake of preventive health services and poorer child growth outcomes.^31^ This study adds to the existing evidence by providing context-specific insights from rural Zimbabwe, thereby expanding the understanding of the risk factors and effects of maternal CMDs in a resource-constrained setting.

Common mental disorder symptoms are characterised by fatigue, impaired concentration, emotional withdrawal and heightened stress reactivity, all of which may reduce a mother’s ability to engage in responsive feeding and consistent caregiving.^32^ Psychological distress may also diminish motivation or confidence to attend health services, contributing to lower ANC attendance and reduced uptake of child health interventions.^33^ In resource-limited contexts such as rural Zimbabwe, these psychological burdens operate alongside structural constraints, amplifying their impact on caregiving capacity.

Higher educational attainment has consistently been reported to be protective against maternal perinatal CMDs.^26,33^ Improved education is often accompanied by greater health literacy, enhanced access to social and economic resources and more effective problem-solving and coping strategies, all of which may buffer women against the onset or persistence of depressive and anxiety symptoms.^34^ Education may also empower mothers to seek timely health care, adopt recommended child-feeding practices and navigate psychosocial stressors more effectively, thereby addressing the adverse effects of socioeconomic disadvantage on maternal mental health.^35^

Conversely, economic insecurity and unemployment are reported as strong correlates of maternal CMDs.^26,36^ A lack of stable income contributes to chronic stress, reduced access to health and social supports, and diminished capacity for responsive caregiving, all of which heighten the risk of depression and anxiety.^37^ Joblessness and limited economic options deplete financial resources and opportunities for survival, which in turn intensify interpersonal stress, cumulatively increasing vulnerability to maternal CMDs.^38^

Household composition, reflected through higher parity or the presence of multiple young children, was associated with increased maternal anxiety symptoms. This finding aligns with existing evidence showing that greater caregiving burden and limited childcare support elevate maternal stress and anxiety levels.^39^ Prior studies have also distinguished the effects of parity on different CMD dimensions, with anxiety often being more sensitive to the practical demands of child-rearing and resource constraints than depression.^40^ The current findings reinforce this distinction, suggesting that cumulative caregiving responsibilities may heighten maternal anxiety through persistent concerns about meeting children’s nutritional, emotional and financial needs.^41,42^

Maternal depression and anxiety can adversely affect caregiving through multiple, interrelated pathways.^43^ Psychologically, CMDs are characterised by diminished emotional responsiveness, fatigue and impaired concentration, which can reduce a mother’s capacity to engage in sensitive and consistent caregiving.^44^ Depressed or anxious mothers may struggle to interpret and respond appropriately to their child’s hunger and distress cues, leading to irregular feeding schedules, reduced dietary diversity and premature cessation of breastfeeding.^45^ Such behaviours can contribute to undernutrition and, in some cases, maladaptive feeding patterns associated with overweight or obesity in early childhood.

Maternal CMDs are often linked to lower uptake of preventive and promotive health services. Depressive symptoms may erode motivation and self-efficacy, leading to missed opportunities for immunisation, growth monitoring and vitamin A supplementation.^44^ Anxiety, on the other hand, may manifest as excessive worry or avoidance of healthcare settings, further limiting access to essential child health interventions.^45^ In resource-limited settings such as rural Zimbabwe, these psychological burdens are often compounded by poverty, food insecurity and limited social support, intensifying the negative cycle between maternal CMDs and child malnutrition.

### Strengths and limitations

By modelling a comprehensive set of household-, maternal- and child-level variables simultaneously, this study provides a more nuanced understanding of the interrelated drivers of maternal CMDs and child nutritional outcomes than many earlier single-factor analyses. The inclusion of both maternal and spousal socioeconomic indicators, child health characteristics and household composition variables allowed a multidimensional exploration of risk and protective factors in the Zimbabwean rural context.

However, several limitations should be acknowledged. The study utilised secondary data derived from a case-control design that was not originally intended to examine maternal CMDs as primary exposures or outcomes. Consequently, some relevant psychosocial or contextual factors that could influence maternal mental health and caregiving behaviours may not have been captured, potentially introducing residual confounding. Additionally, maternal CMDs were assessed at a single time point, limiting the ability to infer temporal or causal relationships. Given that chronic malnutrition, such as stunting, develops cumulatively over time, a cross-sectional measure of maternal mental health may not fully reflect the dynamic and potentially fluctuating nature of CMDs in relation to child growth trajectories.

Moreover, data collection for key historical measures, such as birth weight, height, HIV status and maternal ANC attendance, relied on information extracted from child health cards. This approach, while practical, led to the exclusion of participants without these records, potentially introducing selection bias by disproportionately excluding more socioeconomically disadvantaged households or those with weaker engagement with health services.

While the original dataset was derived from a matched case-control design, matching could not be retained in this secondary analysis, so standard regression methods were applied. Residual confounding remains possible, particularly from unmeasured psychosocial stressors such as intimate partner violence or social support.

Finally, the study assessed CMDs only among mothers of children aged 6 months and older, potentially overlooking the critical perinatal and early infancy periods when maternal mental health exerts the strongest influence on caregiving and nutritional outcomes. Social desirability effect from self-reported sensitive outcomes, such as mental health symptoms and household socioeconomic status, could also result in reporting bias.

## Conclusion

Maternal mental health, household socioeconomic circumstances and child nutritional outcomes are closely linked. The findings emphasise the need for an integrated approach to maternal and child health programming that recognises the deep interconnections between maternal mental health, socioeconomic disadvantage, caregiving practices and child nutritional outcomes. Addressing maternal CMDs within routine maternal and child health services offers an opportunity to simultaneously improve maternal well-being and child growth outcomes. Coupling mental health care with social, economic and nutrition interventions offers a strategic pathway to break the cycle linking maternal distress and childhood malnutrition in Zimbabwe and similar settings.

### Recommendations

The findings underscore the importance of integrating mental health screening and support into routine maternal and child health services. Identifying maternal CMD symptoms at the primary healthcare level could help flag households at increased risk of suboptimal caregiving and child malnutrition. Integrating mental health support with nutrition counselling and social protection interventions may offer a pragmatic approach to addressing the interconnected challenges faced by mothers and children in similar settings. Strengthening intersectoral collaboration between the health, nutrition, social welfare and community development sectors may further enhance the coordination and sustainability of such interventions. Community-based partnerships could also support early identification, referral and continuity of care for vulnerable mothers and children.

To address the limitations of a cross-sectional dataset, conducting longitudinal research will more accurately capture how fluctuations in maternal mental health over time influence child growth trajectories and care practices. In addition, future studies should employ inclusive, diverse data collection methods that capture the experiences of mothers outside the formal health system. Intervention studies that integrate maternal mental health support with nutrition and social protection programming are needed to provide causal evidence of impact.

Randomised and quasi-experimental designs could help determine whether improving maternal psychological well-being translates into sustained improvements in child nutrition and health outcomes. Qualitative research will also be crucial in unpacking cultural conceptions of maternal distress, caregiving expectations and barriers to mental health care, ensuring that interventions are both contextually appropriate and socially acceptable.
